# A Roadmap to Inform the Implementation of Evidence-Based Collaborative Care Interventions in Communities: Insights From the Michigan Mental Health Integration Partnership

**DOI:** 10.3389/fpubh.2021.655999

**Published:** 2021-05-24

**Authors:** Amy Rusch, Lindsay M. DeCamp, Celeste M. Liebrecht, Seo Youn Choi, Gregory W. Dalack, Amy M. Kilbourne, Shawna N. Smith

**Affiliations:** ^1^Department of Health Management and Policy, University of Michigan School of Public Health, Ann Arbor, MI, United States; ^2^Michigan Public Health Institute, Okemos, MI, United States; ^3^Department of Learning Health Sciences, University of Michigan Medical School, Ann Arbor, MI, United States; ^4^Department of Psychiatry, University of Michigan, Ann Arbor, MI, United States; ^5^U.S. Department of Veterans Affairs, Quality Enhancement Research Initiative, Washington, DC, United States; ^6^Institute for Social Research, University of Michigan, Ann Arbor, MI, United States

**Keywords:** implementation roadmap, sustainability, behavioral health services, mental health, evidence-based, Medicaid, collaborative care, implementation science

## Abstract

**Background:** Despite increasing calls for further spread of evidence-based collaborative care interventions (EBIs) in community-based settings, practitioner-driven efforts are often stymied by a lack of experience in addressing barriers to community-based implementation, especially for those not familiar with implementation science. The Michigan Mental Health Integration Partnership (MIP) is a statewide initiative that funds projects that support implementation and uptake of EBIs in community-based settings. MIP also provides an *in situ* implementation laboratory for understanding barriers to the uptake of EBIs across a variety of settings. We report findings from a statewide qualitative study of practitioners involved in MIP projects to garner their perspectives of best practices in the implementation of EBIs.

**Methods:** Twenty-eight semi-structured interviews of practitioners and researchers from six MIP Projects were conducted with individuals implementing various MIP EBI projects across Michigan, including stakeholders from project teams, implementation sites, and the State of Michigan, to identify common barriers, challenges, and implementation strategies deployed by the project teams, with the purpose of informing a set of implementation steps and milestones.

**Results:** Stakeholders identified a number of barriers to and strategies for success, including the need for tailoring program deployment and implementation to specific site needs, development of web-based tools for facilitating program implementation, and the importance of upper-level administration buy-in. Findings informed our resultant community-based Implementation Roadmap, which identifies critical steps across three implementation phases—pre-implementation, implementation, and sustainability—for implementation practitioners to use in their EBI implementation efforts.

**Conclusion:** Implementation practitioners interested in community-based EBI implementation often lack access to operationalized implementation “steps” or “best practices” that can facilitate successful uptake and evaluation. Our community-informed MIP Implementation Roadmap, offering generalized steps for reaching successful implementation, uses experiences from a diverse set of MIP teams to guide practitioners through the practices necessary for scaling up EBIs in community-based settings over pre-implementation, implementation and sustainability phases.

## Introduction

Innovative and effective evidence-based collaborative care interventions (EBIs) for addressing behavioral health concerns are being developed to serve a variety of populations and settings (1, 2). However, the creation of EBIs does not necessarily translate into their implementation by practitioners (2–5). Often practitioners looking to implement new EBIs are stymied by lack of knowledge or understanding of how to identify and address barriers to community-based implementation (6). In order to expand the use of EBIs, implementation scientists can help fill this gap by developing and disseminating tools that can support, direct, and protocolize EBI implementation efforts by community-based practitioners.

An Implementation Roadmap that helps practitioners define and address challenges in implementing EBIs provides one potential, readily disseminatable tool for supporting EBI implementation. Implementation Roadmaps, similar to Roadmaps developed in organizational studies and business communities [e.g., Technology Roadmaps (7)], provide “scripts” for the critical steps practitioners should follow in scaling EBIs to new settings (8–11). Established EBIs can include standardized implementation steps and implementation resources that can support generalized EBI implementation, but few models provide specific steps and pathways to implementation across a variety of real-world settings (9–12). Implementation Roadmaps can guide process development by highlighting key steps and recommending “best practices” for use throughout the real-world implementation process. Implementation Roadmaps aim to increase the efficiency of real-world implementation efforts by outlining, in advance, the different stages of the implementation process and identifying the actions practitioners might want to take to anticipate, accommodate, and/or alleviate barriers to successful EBI deployment. The Quality Enhancement Research Initiative (QUERI) Implementation Roadmap illustrates how a Roadmap can act as a researcher-community implementation guide, demonstrating the utility of this tool (13). A well-designed Implementation Roadmap can help bridge the gap between implementation scientists and practitioners by leveraging implementation science expertise to help demystify the implementation process (14–16).

To support implementation of interventions, implementation scientists have developed numerous frameworks for describing, understanding, and evaluating the implementation process. Frameworks such as the Consolidated Framework for Implementation Research (CFIR) (17), Exploration, Preparation, Implementation, Sustainment (EPIS) (18), and Reach, Effectiveness, Adoption, Implementation, and Maintenance (RE-AIM) (19), among others, have been created to help implementation scientists understand and conceptualize key aspects of implementation processes (20–23). While these frameworks have done a great deal to push forward the science of implementation, their extant differences in definitions and terminologies, use of field-specific and often technical language, and generalist scope can limit their ability to directly inform implementation efforts for (non-implementation scientist) front-line practitioners.

Implementation scientists have also developed specific implementation strategies, or theory-based “methods or techniques used to enhance the adoption, implementation, and sustainability of a clinical program or practice” to aid in implementation (24, 25). For example, the Expert Recommendations for Implementing Change (ERIC) project systematically refined a compilation of implementation strategies for implementation science (24). We see the MIP Implementation Roadmap as a tool that can complement these implementation strategies by helping community-based implementers systematically plan out their implementation efforts, including identifying and deploying the implementation strategy or strategies most appropriate for their project and community setting or settings.

The goal of this study was to create an Implementation Roadmap based on the experiences of a structured partnership between research institutions, community mental health clinics/centers/agencies, and the State of Michigan to assist investigators implementing novel EBIs in community settings across Michigan. Through this Implementation Roadmap, practitioners in this partnership are guided through the steps of implementation in community settings based on experiences of other projects and implementation science best practices.

### The Michigan Mental Health Integration Partnership

The Michigan Mental Health Integration Partnership (MIP) is a collaboration between the Michigan Department of Health and Human Services (MDHHS) and the University of Michigan (UM) with a goal of providing administrative support to enhance services and delivery of integrated care for children and adults with behavioral health care needs that are served by Michigan's Medicaid program. MIP-associated projects link clinical experts working with MDHHS and Michigan communities to implement effective practices for depression, bipolar disorders, and other mental or behavioral disorders to enhance the quality of care and well-being of low-income citizens in Michigan. This partnership offers a structured program to expand access to novel EBIs addressing behavioral health issues and help reach populations with behavioral healthcare needs who might not receive them otherwise. As demand for Medicaid services expand, providers are increasingly strained, which impacts organizations' ability to participate in the uptake of EBIs; MIP seeks to target barriers to increase EBI use in these settings (26). Since its inception in 2006, MIP has been instrumental in deploying physical and mental health integrated care in Michigan's Community Mental Health Programs. In fiscal year 2019 (FY19), 22 projects in 80 out of 83 Michigan counties were supported by MIP.

In addition to advancing evidence-based care for Michiganders with behavioral healthcare needs, MIP provides a real-time laboratory to explore implementation processes in community-based settings. A small proportion of the MIP administrative budget every year supports implementation scientists that advise investigators on their implementation efforts and inform the development of tools to help MIP investigators. As MIP projects are funded one year at a time, there is relatively little time to train and orient sites to implementation practices. Additionally, most MIP investigators are not implementation scientists, but clinicians and practitioners bringing their experience and expertise to implement EBIs in community settings. They often lack either experience in community-based implementation or exposure to implementation science principles. The MIP Administrative Team opted to develop an Implementation Roadmap that laid out necessary steps and “best practices” that could be easily adopted by MIP investigators to inform their implementation projects. Having MIP investigators as the immediate target audience for the Implementation Roadmap, the team opted to use experience of recent MIP projects to inform the Roadmap.

## Materials and Methods

### Participant Selection

MIP projects active during FY19 (*n* = 22) comprised our study population. While all projects were invited to participate, our team also reached out to MIP project teams individually to request participation. In line with our intent to specifically define “best practices” for the Roadmap, we sought out project teams that were known to have had success in implementing their projects (or had seen significant EBI adoption) in one or more community-based settings, as identified from insights shared by the Partnership Director. For all projects that participated, we aimed to interview relevant MIP project personnel (i.e., Principal investigator [PI], project managers, key investigative/operational staff) as well as at least one key stakeholder at the community implementation site(s). Project PIs were initially approached about participating, and then were asked to identify key project staff. Upon completion of study team interviews, interviewees identified select community partners that were instrumental in their implementation project and available for further interviews. To ensure representation of sites, study teams active at more than one site were asked to identify both “early adopters” (27) (or community partners that had few issues implementing the EBI) as well as partners that experienced more barriers to implementation. Our team then contacted these partners for interviews. We continued conducting interviews with new MIP teams until thematic saturation of research questions was reached, both within and across MIP project teams. Saturation was assessed by the interviewer during data collection based on a dearth of new information appearing in interviews. As MIP is designed to inform statewide policy related to behavioral health, we also reached out to key MDHHS personnel for interviews to represent the state policy lens, especially on matters related to statewide diffusion and long-term sustainability of new EBIs.

### Interview Methodology

Interviews were semi-structured, informed by an interview guide that was developed by the MIP Administrative Team, and tailored to each group of participants based on their role in the implementation process (e.g., PI, community partner) and information shared in previous project interviews. The interview guide was structured around three pre-specified phases of implementation, based on the Replicating Effective Programs (REP) framework, which we anticipated would form the basis of the Implementation Roadmap: pre-implementation, implementation, and sustainability (28). All interview questions followed the same structure around the three specific implementation phases as defined by REP, but follow-up prompts were tailored based on the interviewee's role to increase the efficiency of interviews and ensure appropriate detail for phases that were most applicable to the interviewee. Interviews were generally 60 minutes in length and were conducted in-person or over the phone when requested. All interviews obtained verbal consent and were digitally recorded. Interviews were generally one-on-one, but on three occasions teams requested to have multiple project staff interviewed together. Interviews were conducted May-December 2018 and no compensation was provided for interviews. The Consolidated Criteria for Reporting Qualitative Research (CORE-Q) guided this project. This project was reviewed and considered by the University of Michigan Institutional Review Board (IRB) to be non-regulated and exempt from further IRB review.

### Data Analysis

Following the interviews, recordings were verified by the interviewer and transcribed verbatim. An inductive, iterative process identified emergent themes across interviews, with a focus on key input for the Roadmap—notably identifying the successful strategies deployed by MIP projects during implementation, implementation barriers encountered, and the lessons learned from study teams and site partners. Each interview was independently analyzed at the individual level, with the interviewer generating the initial definition for all themes, and then all investigators on the MIP Administrative Team generated a consensus codebook. As the MIP Administrative Team used a heuristic approach to Roadmap development, the codebook was reviewed after every 2–3 interviews to assess emergent categories not captured in earlier iterations. NVivo 12 was used for all data management and coding. All transcripts were coded by one experienced individual with graduate training in qualitative methods who has contributed to several prior qualitative, interview-based research projects.

Interview codes captured common tactics deployed, successful steps taken, barriers faced, and missteps experienced by projects and identified through interviews. Codes were compared across interviews and projects and between MIP project study staff, community partners, and MDHHS staff. Codes were also compared across projects to ensure that findings were reflective of multiple (if not all) projects, and not solely reflective of interviews representing only one project. The resultant findings were combined to identify “best practices” that were either used or retrospectively desired in EBI implementation across MIP projects. “Best practices” were then grouped to align with the three phases of implementation identified from the updated REP framework: pre-implementation, implementation, and sustainability. Within each implementation phase, generalized stages were identified by grouping “best practices” based on tactics and experiences shared by interviewed teams; these subcategories and their content comprised the MIP Implementation Roadmap.

## Results

Six MIP projects, out of 22, opted to participate in interviews for Roadmap development, with five of these projects recognized as having shepherded successful community-based implementation at the time of interview (Table 1). *N* = 34 individuals representing these six projects were contacted for study interviews, and *N* = 28 (82%) completed interviews. Due to study constraints and differing stages of the projects' implementation progress, not all personnel involved with each project's implementation effort were identified or invited to participate in interviews; rather, we focused recruitment efforts on ensuring representation of at least one of each key stakeholder group (PI, project manager or study staff, community-based implementation partners) for each project.

**Table 1:** Projects (EBIs) interviewed.

**Project goals**	**Professionals delivering EBI**	**Primary EBI target population**	**Service delivery sites**	**Data providers informing MIP Roadmap**	**MIP Roadmap phase at time of interview**
Implement and evaluate a 3-tiered model of behavioral health programming that will provide evidence-based mental health prevention-to-intervention services to schools	Trained School Professionals	Youth	School	PI, Project Manager, Community Partners (School Professionals)	Implementation
Provide services including just-in-time phone consultation to primary care providers and telepsychiatry consultation to youth; Expand the roles of embedded Behavioral Health Consultants to increase access to mental health treatment for underserved youth and high-risk perinatal women	Primary Care Providers	Youth	Hospital/Clinic	PI, Project Manager, Project Staff, Community Partners (Behavioral Health Consultants)	Implementation
Develop and implement a program for high-risk fathers to support family engagement through parenting and family support interventions, activities for school and work re-integration, and family service connections	Family Service Employees	Adult	Family Service Center	PI, Project Manager	Pre-implementation
Develop standard protocols and reporting mechanisms for medical and mental health care use and quality of care among CMH clients from the county using real-time and population-based data to build a care bridge	County Agency Providers (multiple roles)	Adult	Community Mental Health Agency	PI, Community Partner (Deputy Director)	Implementation
Develop and implement a program delivered through obstetric clinics for high-risk women who are preconception, pregnant or between pregnancies to promote positive maternal-fetal/infant outcomes	Obstetric Providers	Adult	Hospital/Clinic	PI, Project Manager, Community Partner (Doctor, Clinical Coordinator)	Implementation
Build and evaluate a Collaborative Care Implementation and Support Team that will train and provide technical assistance to Community Health Centers to implement the collaborative care model	Community Health Care Providers	Adult	Community Health Center	PI, Project Manager, Project Staff, Community Partners (Clinical Director, Quality Improvement Coordinator)	Implementation

Of the 28 interviews, *N* = 14 (50%) were with PIs, project managers, and study staff from the six MIP study teams. All six projects provided name(s) of community-based implementation partner(s). *N* = 11 (39%) community partner interviews were completed with key stakeholders at these sites, with at least one site represented for five of the six projects. Five community partners who were identified by MIP study teams for interviewing purposes declined participation. Additionally, *N* = 3 (11%) interviews were conducted with MDHHS staff; all contacted MDHHS staff agreed to participate. *N* = 23 (82%) interviews were completed in-person, while *N* = 5 (18%) were completed over the phone by request (*N* = 3 MDHHS staff and *N* = 2 MIP project staff) (Table 2).

**Table 2:** Stakeholders interviewed.

	**Project teams**	**Community partners**	**MDHHS staff**
Definition	Project team interviews included PIs, project managers, and other staff key to the implementation process	Community partners were key personnel at the implementation site projects were implementing their projects at and assisted in implementation efforts	Interviewed staff members at MDHHS are part of the MIP Partnership and review/approve MIP projects
# of interviews (*N* = 28)	14	11	3
Projects represented (*n* = 22)^a^	6	6	NA

a *Not all projects contacted for interviews, Six projects ultimately comprised the study sample*.

### Informing the Roadmap

**Figure 1:**
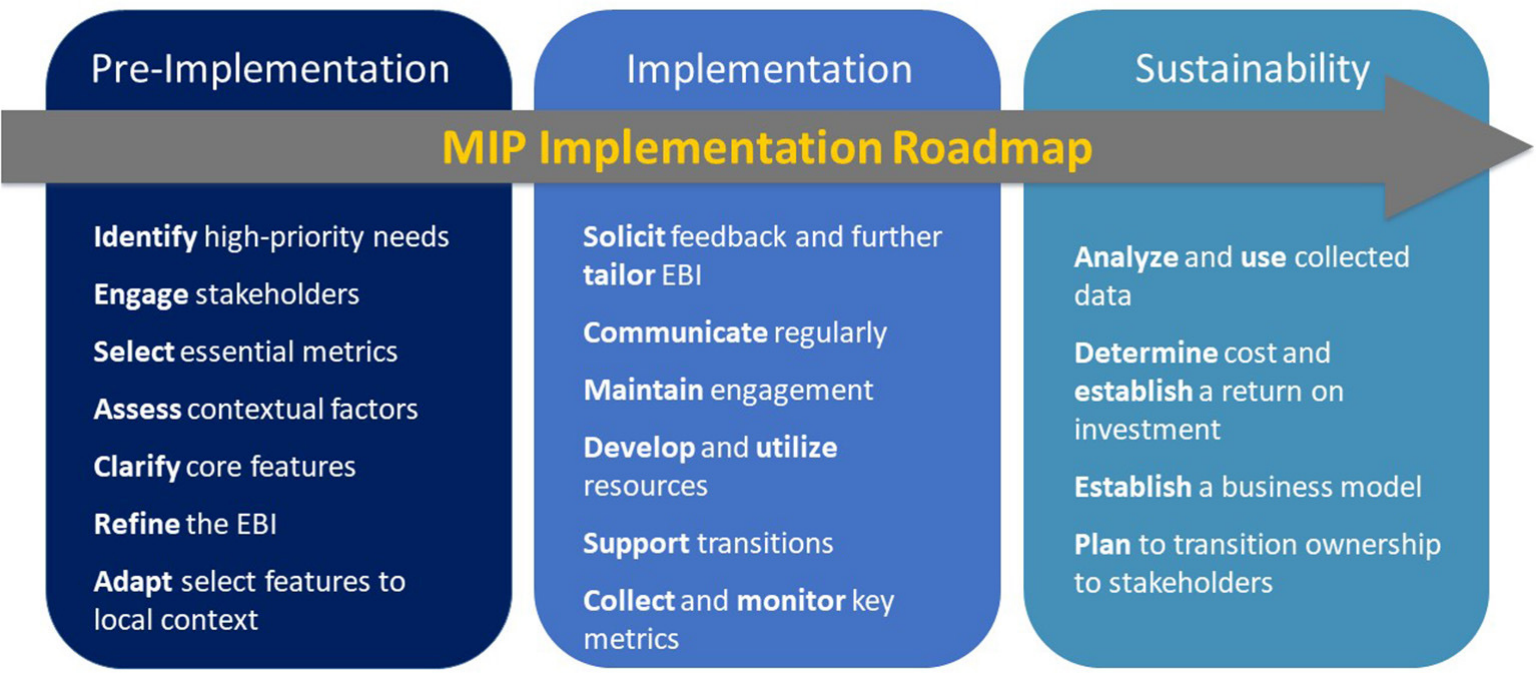
MIP Implementation Roadmap.

Our analyses identified “best practices,” which we then mapped onto the three phases of the REP framework and used to identify actionable steps implementers could take to help anticipate, accommodate, or alleviate implementation barriers (Figure 1, Table 3). Within each implementation phase, we present the steps identified and results from analyses explaining how they advance implementation.

**Table 3:** Implementation Roadmap components.

**Implementation phase**	**Implementation Roadmap component**	**Example actions identified by interviewees**
Pre-implementation	Identify high priority needs	• Disconnect between the needs of community and the needs of research project can impact implementation success. Align needs from the start. *[PI]* • Performing a formal needs assessment on-site can ensure that stakeholder-communicated needs were consistent with observed “on the ground” needs and that the EBI aligned with the specific, high-priority needs of the community. *[Project Staff]*
	Engage stakeholders	• Bring together diverse representatives from all constituents and stakeholders from the beginning. Regular meetings with frontline “day-to-day” practitioner and implementers from the start ensures ongoing investment. *[PI/Project Staff]* • Identify a project champion who can advocate for EBI use when the implementation project team was not physically present. *[PI]*
	Select essential metrics	• Take time to develop organization/reporting systems from the start. Plan for their usage throughout the implementation and sustainability phases of a project. *[Project Staff/Community Partners]* • Sample metrics to consider collecting data on and monitoring include the number of providers trained, number of consumers receiving EBI, counties served, etc. *[PI/Project Staff]*
	Assess contextual factors	• On-site staff do not necessarily know what leadership is agreeing to, so webinars/calls/meetings with these staff members can help minimize miscommunication and confusion from the start. *[Project Staff]* • Time-intensive tasks often fall outside of the immediate research teams' control, and the research teams, community partners, and MDHHS all operate under different time constraints; assessing and budgeting for these differences in timelines can lead to better EBI implementation. *[Project Staff/Community Partners]* • Collaborate with partners to identify/plan for all contextual factors that could impact EBI implementation and establish norms for revisiting these plans if/when other factors arise. *[PI]*
	Clarify core features of the EBI	• Talk about practical components, i.e., explicitly what the project will look like, space needs, staffing needs, technology availability, and roll out plan, initially. *[Project Staff/Community Partners]* • Walk through a process mapping exercise with partners for EBI implementation before implementation begins. *[Project Staff/Community Partners]*
	Refine the EBI	• Do a needs assessment. Spend time at sites to see the needs in the context of the community setting, if viable. *[Project Staff/Community Partners]* • Remain open to refinements throughout the pre-implementation process and encourage community partner participation and feedback in this step. *[Project Staff]*
	Adapt select features to local context	• Send team members physically to sites to understand the local context. Workflows vary by clinics so allow for workflows to be tailored to local circumstances. *[Project Staff]*
Implementation	Solicit feedback and further tailor EBI	• Solicit feedback frequently and adapt as needed via emails, polls, phone calls or visits. Gather feedback from different levels of the organization. *[Project Staff]*
	Communicate regularly	• Maintain face-to-face meetings when possible, following up on relationship building from pre-implementation. Maintain site engagement through electronic tools and online spaces to share experiences and feedback both between community sites and research teams as well as among community sites. Share expertise and findings when possible and appropriate. *[PI/Project Staff]*
	Maintain engagement	• Timing between recruitment for and initiation of an EBI can be long and lead to engagement struggles. Once started, regional support groups are good opportunities for growing teams to report to each other and continue conversations as teams spread across the state. *[Project Staff]*
	Develop and utilize resources	• Invest in high quality, useful material. Monitor the needs of community partners and use the implementation process to develop needed resources that can be used long-term by community sites. Consider developing technological-based tools and resources that can be tailored to sites. *[Project Staff]*
	Support transitions	• Leadership transitions are unpredictable and hard to prepare for, so work with partners early when a leadership change is announced. Community sites can experience higher staff turnover, so develop a plan to maintain implementation should staff changes occur. *[PI/Project Staff]*
	Collect and monitor key metrics	• Be upfront that documentation and reporting takes time. Reporting requirements can get burdensome, so be upfront about the process for data collection and monitoring as it begins. Plan for opportunities to share collected data and findings with community partners throughout the implementation process. *[Project Staff/Community Partners]*
Sustainability	Analyze and use collected data	• Showing evidence of efficiency and effectiveness and fidelity allows projects to expand. Analyze collected metrics and inform stakeholders of project impacts. *[PI/Project Staff]*
	Determine costs and establish a return on investment	• Compensate providers/implementers for their time whenever possible. Consider their time as part of your costs. Consider outcomes impacted by EBI implementation beyond primary health outcomes; include outcomes as part of projects' return on investment. *[PI]*
	Establish a business model	• Ability to fund staff working on a project beyond the year(s) of funding is problematic for some sites. Consider solutions before funding is removed. Utilize partnerships with MDHHS to carry out conversations with policymakers/funders on long-term funding mechanisms. *[PI]*
	Plan to transition ownership to stakeholders	• Think about the policy implications of the work being done/carried out and how that can be used to influence sustainability from a policy perspective. This can often be incentivizing for sites. *[PI]*

### Pre-implementation

Generally, interviewees recognized that preparatory work prior to any active implementation effort was fundamental to successful implementation. However, even among the successful MIP implementation teams, both study staff and community partners recognized that more time and effort could have been used to prepare. Multiple MIP study teams recommended budgeting at least double the amount of time originally estimated for their preparation stage. To guide the pre-implementation phase, summarized in Table 4, we identified seven steps for implementers to take: (1) identifying high-priority needs; (2) engaging stakeholders; (3) selecting essential metrics; (4) assessing contextual factors; (5) clarifying core features of the EBI; (6) refining the EBI; and (7) adapting select features to local contexts.

**Table 4:** Pre-implementation illustrative quotes.

**Pre-implementation steps**	**Supporting quotations from interviews**
Identify high-priority needs	“Our project was definitely not a solution looking for a problem, it was the other way around.” [Project Staff Member]
Engage stakeholders	“One thing I learned earlier on was to really engage all the right people at the table. We were told, ‘These are the right people to talk to. This doctor, that person…' and it turned out while they were important people, they were not the right people.” [PI] “Engaging leadership will get you in the door, but engaging opinion leaders will keep you in the house.” [PI] “It's building the relationships, making sure that everyone is involved from the get go. Really valuing everyone's contributions but also always thinking about, I know what I need out of this, but that might be different than another one of the stakeholders needs to get out of this and how do we make sure that we're addressing all of those things.” [PI] “To build rapport and make our program a reality we needed to go in person, bring food, and have those meetings.” [Project Manager]
Select essential metrics	“Since our project is still kind of in the beginning stages, we are being thoughtful as far as data collection. We do have a statistician who specializes in cost effectiveness because it's not something that in general we have, those data analyses we'd like to do is kind of its own thing. So just even thinking ahead of time, if we're gonna want to show that, what do we need data wise to be able to do that.” [PI] “You don't want to just do something for the research so we actually involved [community sites] in choosing measures when we did trainings for all of their clinicians.” [Project Staff Member]
Assess contextual factors	“Our team started by thinking through every aspect of how this might work and what we need to do to figure this out. Do we have psychiatrists that are available? Are we gonna cover their effort? What does their day look like? How are they gonna answer the phone? How's the call gonna come to them? How's the information gonna come to them? What information do we need to gather? We just started digging into the details for every component.” [Project Manager]
Clarify core features of the EBI	“While initial conversations about the project went really, really well, 6 months down the road when we're implementing we found out that those conversations didn't trickle down to the right people.and it's almost like restarting and reselling all of the reasons that we're doing this.” [Project Staff Member]
Refine the EBI	“I think keeping in mind what the need of the community is rather than the need of the program, that's really critical. if you build a program that's responsive to that need, it's gonna be adopted and utilized widely. If you build a program that fits your need as a researcher, but doesn't fit a need in that community, it's not gonna be used.” [PI] “Programs need to be low barrier. and very user friendly for those delivering [the EBI] in order for our community sites to use new practices.” [Project Manager]
Adapt select features to local context	“I really struggled with workflow. In the beginning there were a lot of questions of, you know ‘how would we roll this out' ‘what would our process be' and we couldn't really answer those questions without knowing, ‘what does this entail' ‘what is the workflow'.” [Community Partner] A workflow is “a couple pages that highlighted from the very beginning what to expect all the way toward the end. It helped us build a plan to know what our staff would do. and that helped a ton.” [Community Partner] “So, you know, it's really a one size does not fit all thing. So you're trying to get the program to go, but you have to tailor it a little bit to the local circumstances.” [PI] “Initially we were struggling with the planning process for bringing a new intervention to a new site, but once we were able to do a workflow analysis, we were able to understand the adjustments that needed to be made.” [Community Partner]

#### Identify High-Priority Needs

An early pre-implementation step for all MIP projects was the identification of needs and priorities within the communities they planned to serve. Several teams communicated that a mismatch between the needs of the community and the needs of the project could curtail implementation efforts. MIP teams encouraged aligning these needs from very early on in the implementation process as a precursor to successful implementation.

#### Engage Stakeholders

Nearly every interviewee identified stakeholder engagement as crucial to ensuring successful EBI implementation. Especially in larger organizations, ensuring that all stakeholders were on board with the project from the beginning decreased the time spent on other pre-implementation steps. Leadership buy-in was cited repeatedly, but teams also stressed the importance of bottom-up engagement. MIP Teams emphasized the importance of regular, in-person or videoconference meetings with site stakeholders, ideally with the PI present, as a way to both increase stakeholder engagement and reassure sites that study teams were invested in the EBI's success.

#### Select Essential Metrics

Study team members said early metric selection was important for measuring progress during the implementation process, but also helpful for engaging stakeholders and ensuring similar goals and expectations for the project across stakeholders during pre-implementation. Project teams and investigators recognized the importance of selecting and measuring baseline values for key metrics prior to any implementation effort for informing future sustainability. For example, accurate baseline measures regarding provider use and fidelity of the EBI, as well as receipt, quality of care, and outcomes among consumers who received or did not receive the EBI, are important to determine the impact of EBI implementation.

#### Assess Contextual Factors

In line with frameworks like the CFIR, assessing the context allowed study teams to identify potential barriers or facilitators to their proposed project. Typically, the contextual factors identified by teams were those defined by the CFIR as “inner setting” characteristics (17): for example, anticipated employee time/effort available to implement the EBI, availability of protected employee time to carry out EBI implementation, current or anticipated staffing shortfalls, physical space availability, technological capacity (e.g., Electronic Health Records), and/or internal site regulatory processes.

#### Clarify Core Features of the EBI

Several successful projects noted that once stakeholders were engaged and the EBI was deemed appropriate, it was critical to ensure that all stakeholders understood the core features of the EBI *prior to the beginning of active implementation*. Study teams highlighted the importance of talking with the community stakeholders about both the scientific and practical components of the implementation effort, including outlining the project timeline and steps in the implementation process (e.g., opportunities for training, consumer follow-ups) as well as being very explicit about expectations for logistical needs, such as staffing, effort, and space. Explicitly clarifying features for all involved is important, especially when prioritizing efficiency, and necessary for completing MIP objectives in a limited time.

#### Refine the EBI

As is the case with most investigator-led implementation efforts, MIP teams all had an EBI in mind that they planned to implement—and indeed were funded to implement a specific EBI. Nonetheless, teams still reported that assessing the fit between their selected EBI and the identified high-priority need(s) of the site(s) was a key step. Successful projects noted that they remained open-minded about potential refinements of their intervention to ensure that implementation would be feasible in their community setting. Refining an intervention to fit the needs and setting, while ensuring preservation of essential evidence-based elements, is a key step in allowing implementation to move forward. Note that refinement of the EBI may take a couple of iterations, such as through the use of rapid-cycle testing of the EBI implementation, especially as additional barriers are discovered through assessment of contextual factors.

#### Adapt Select Features to Local Context

Core feature identification, EBI refinement, and contextual factor assessment all helped study teams to adapt their selected EBI to their local site's context. Nearly all projects we spoke with mentioned that some kind of adaptation had to be done, but not all sites had planned for adaptation prior to active implementation. Among several documented ways to plan for local adaptation, one that emerged from our interviews was workflow analysis—a process in which project staff and community partners mapped the sequence of tasks to produce a resultant implementation outcome for specific community sites. The workflow analysis process, an essential way to understand the EBI core elements and to identify adaptation or menu options to fit the local context (e.g., via rapid cycle testing), helped the study teams to lay out a step-by-step plan detailing how their project would be carried out with their community partners. This process proved especially helpful to MIP teams implementing their EBI in multiple community settings, as it allowed them to systematically consider between-site differences, as well as concomitant differences in adaptations necessary to accommodate the variations. Another way that many teams adapted their EBI was by tailoring projects to local context based on feedback from community sites.

### Implementation

Common practices to promote EBI implementation emerged across teams. From these commonalities, we identified six steps for the implementation phase (see Table 5): (1) soliciting feedback for further EBI tailoring; (2) communicating regularly; (3) maintaining engagement; (4) developing and utilizing resources; (5) supporting transitions; and (6) collecting and monitoring key metrics.

**Table 5:** Implementation illustrative quotes.

**Implementation steps**	**Supporting quotations from interviews**
Solicit feedback and further tailor the EBI	To solicit regular feedback “we built in opportunities for direct feedback from participants every week in the beginning” so they could make weekly EBI adjustments in real time. This team “ended up retaining [weekly feedback opportunities] because.it ended up being a useful tool in more ways than [they] anticipated.” [Project Manager] “I definitely took note of how vastly different each of our sites are. In terms of their structure, their resources, even the providers that they have, their culture, their climate, each site is just very very different and I think our team learned very quickly and benefitted from our flexibility in approaching them uniquely.” [Project Team Member]
Communicate regularly	Teams develop a varied communication strategy; for example, “we send out messages about every 6 months saying ‘Hey, our project is still up and going. We look forward to hearing from you!' [They] send some new cards or other resources by mail or electronically. Our PI sends an update around the holidays and in the spring about the progress of the program.” [Project Staff Member] “It drives me absolutely crazy when I get some notice a few days before something is due or if it's like a big project the week before. With this program we always get adequate notice, reminders, so that's really appreciated.” [Community Partner] “That commitment to meet monthly and talk through [issues], is a big reason why this project is still going.” [Community Partner] “One way we learned to modify our transparency with site leadership was by sending out newsletters to all of the sites that have participated to give them feedback even before papers are published.” [PI]
Maintain engagement	“We needed to send frequent reminders to providers about the EBI because.at first you're the fun new thing and then they forget to keep you in the top of their tool box.” [PI] “So I always tell new people.you just have to be persistent. I call it patiently persistent. And then you have to strongly believe in what you're advocating for.” [Project Manager]
Develop and utilize resources	“We [.] are developing resources and materials and adapting them to feedback. We are continuing to grow and enhance the website over time and right now a lot of sites are focusing more on implementation resources provided.” [Project Team Member] “We are using matching funds to develop a website that has been pretty critical in our training for all of our community health centers. We've basically been creating an online manual for both the implementation tasks for health centers as well as the trainings. We also use video webinars and things like that for people to do self-based trainings now. We're building our technology up by the day. And at this point, if you don't have a website you're not a real thing.” [Project Team Member] “I think really creating a program that has high quality, useful materials, that will help sustain fidelity to the original seed that you wanted to disseminate. That feels really critical to me.” [PI]
Support transitions	“When we have a site where there's only one person that participates, that person could get laid off or move or lose interest or not feel like they have sufficient time, and then the program can't continue.” [PI] “Sometimes conversations go really, really well and then 6 months down the road when you're implementing you find out that those conversations didn't trickle down to the right people or those people are gone so you're almost like retraining and reselling all of the reasons that we're doing this if you don't prepare for that.” [Project Manager]
Collect and monitor ongoing metrics	“What is better? What has better outcomes? Where are the providers more satisfied? And also what is more cost effective or if it is more expensive, is it worth the investment?” [Project Team Members]

#### Solicit Feedback and Further Tailor the EBI

During active implementation, MIP teams described seeking feedback from community sites and tailoring their program accordingly. Teams found that their method of implementation often needed to be tailored to particular site needs mid-course during the implementation effort, such as accommodating new barriers to implementation or adjusting to leadership changes. Although tailoring done in the pre-implementation phase was important, further tailoring during active implementation, including course corrections and re-tailoring, allowed teams to readily respond to unanticipated barriers and between-site differences.

#### Communicate Regularly

Regular communication, consisting of high-quality content delivered at manageable frequencies, was important for bringing an EBI to a community setting and maintaining its implementation. Best regular communication practices were not limited to content and timing alone, but also included thoughtfulness and efficiency in communication. Engaging in regular communication while not overburdening partners is a fine line for teams to walk. Steps to reduce the communication burden on community sites, such as minimizing the number of reporting documents required or giving ample notice when reports are due, enhanced effectiveness of communication. Most importantly, teams highlighted communication regularity in maintaining connection between partners through all three stages of implementation.

#### Maintain Engagement

Maintaining provider engagement with the EBI was frequently noted as one of the more challenging aspects of implementation. Sending reminders was one frequently cited tactic, while PI's on-site presence and face-to-face meetings were described as effective ways to maintain engagement. When on-site time was not possible, teams reported maintaining site engagement through electronic tools and online spaces to share experiences and feedback. Others reported maintaining communication between sites in similar geographic areas so that on-the-ground implementers could share their experiences with the project and engage beyond the MIP project team, further creating space for engagement.

#### Develop and Utilize Resources

Improving resource utilization, for example through the development and adaptation of high-quality resources related to the EBI (e.g., training manuals, websites, etc.) that community sites could easily access, was an important implementation step for many teams. Technology development around project delivery was cited by project teams as another resource that aided their implementation work. Community partners viewed technology resources as exciting and useful tools for them, especially when the introduced technology was easy to access and user friendly.

#### Support Transitions

Teams noted that supporting participating staff's transitions was important, as many of their community sites experienced high turnover both at the provider and leadership level. While reflecting on differences in implementation across sites, project teams shared sites with multiple participants were much more likely to allow projects to continue through transitions. Teams noted that with high turnover, and without a plan to introduce and transition the MIP project to the new staff member, the project was left behind without clear steps for onboarding new staff; study teams felt like they needed to start again at square one.

#### Collect and Monitor Ongoing Metrics

Collecting and monitoring identified key metrics was helpful to both aid implementation and prepare for sustainability. This included data on EBI use and quality, consumer outcomes, implementation fidelity, and provider engagement. In particular, fidelity metrics were used to ensure that MIP projects could be applied with fidelity in their chosen setting, which in turn helped to overcome recognized implementation barriers and to support successful adoption of the EBI. Teams noted that they maintained various tools to ensure the fidelity of their implementation strategies throughout the entire implementation effort, including developing a fidelity tool, selecting specific components to monitor implementation, and using self-ratings and/or observed ratings for fidelity.

### Sustainability

To sustain MIP projects beyond initial implementation efforts, MIP teams benefited from having a sustainability plan in place to guide them throughout the implementation process. While plans varied by teams based on funding sources, long-term goals, and other team-specific factors, many teams took similar steps to move their projects to a sustainable model. For the sustainability phase of the Roadmap, we identified four steps for implementers to take as summarized in Table 6: (1) analyzing and using collected data; (2) determining costs and establishing a return on investment; (3) establishing a business model; and (4) planning to transition ownership to stakeholders.

**Table 6:** Sustainability illustrative quotes.

**Sustainability steps**	**Supporting quotations from interviews**
Analyze and use collected data	“When we talked with [stakeholder], it was clear that they're invested in the sustainability of the model.they were interested in our mental health measures or parenting stress measures, and we felt the need to make these [data] clinically meaningful.” [Project Team Member] “I think the project helped open our eyes to some of the other things that maybe we didn't even realize were successes or good outcomes in the work that we were doing. Like being able to really notice increased parental reflection functioning or things that had been changing but we maybe didn't know to call it that or look for that without the study data.” [Community Partner] “The fact that we were able throughout this to develop evidence for efficacy and effectiveness and that we were able to develop a fidelity tool, a training, to sort of evaluate the training, etc., has prepared us now to really take this national.” [Project Team Member]
Determine costs and establish return-on-investment	“The providers that find [the EBIs] valuable use them and the providers that don't necessarily find them having as much value, don't use them in the long run.” [Community Partner]
Establish a business model	“For any program that wants to provide a service to a community that has no funding, without any internal or reliable funding, that's hard.” [PI] “Most programs do not want to be reliant on donor or foundation funding and match funding longer term; it's not very viable for long term sustainment. One path to sustainability is through development of a business model.” [Project Manager] “Although it would be ideal to have an established business model, the research teams participating in MIP projects often do not have individuals with a business expertise working with them directly. The people that are building the programs, myself included, often have no background or expertise in mental health economics, or business modeling or don't have MBA's and so somebody on the team whose role was to say let me help you think about how you could generate revenue from this would be a great addition for probably many programs.” [PI] “Typically, when we're working with a community partner they'll say, ‘we love this, but if we don't have a billing code to sustain it once the grant funding is gone, we can't keep doing it'.” [PI]
Plan to transition ownership to stakeholders	“We are not even applying for an extension of the Medicaid Match project any more. This is it. And it doesn't mean that the project is going to end. I think our partnership [with stakeholders] is so strong that we don't necessarily need it to be a formalized Medicaid match project, because the community site is doing a lot of the things that the study team set out to do.” [PI]

#### Analyze and Use Collected Data

The data collected by MIP project teams throughout their EBI implementation were the most useful tools they had to advocate for resources to help promote the sustainability of their projects. Teams utilized data to show stakeholders the impact of their programs. Analyzing collected data was also a benefit for the teams themselves by highlighting successes and positive outcomes. Sharing and presenting results from data collected throughout the implementation process helped the teams to advocate for the sustainability of MIP projects while also allowing teams themselves to reflect on, show progress, and continue improving the implementation of their EBI.

#### Determine Costs and Establish Return-on-Investment

Replicating, scaling, and maintaining EBI implementation requires a determination of costs. Questions around costs are often one of the first factors that site leadership (or policy makers) consider when evaluating projects' impacts (29, 30). This includes not just the costs to support the EBI directly, but also staffing time for practitioners delivering the EBI and program oversight, technological costs, physical resources, and costs for documenting any key metrics or other EBI information. Exact inputs will vary across programs, and implementers should take time to consider these inputs when determining their program costs.

An important step in moving toward sustainability is establishing the return-on-investment for community-implemented projects. The ability to demonstrate to stakeholders that the resources necessary to start and maintain the EBI can pay dividends (e.g., improved clinical outcomes, increased community throughput, decreased employee turnover or burnout) can improve the potential for both scaling and sustaining EBI implementation efforts (31–33).

#### Establish a Business Model

Implementation projects' ultimate goal is to have a model where projects are not tied to MIP funding and can sustain independently of short-term mechanisms (e.g., grant funding). For many MIP projects, the biggest barrier to sustainability was a lack of billing codes for the practice. MIP's partnership with MDHHS provided access to key policymakers that could help implementers pursue changes to billing codes (34); however, metrics related to cost and effectiveness were necessary to motivate policy change. Establishing a business model for the EBI that includes information on the costs, return-on-investment, as well as the relationships needed to move a sustainable model forward furthers implementation efforts by showing how projects can exist outside of the MIP/Medicaid funding model. In many cases, however, the knowledge necessary to develop a strong business model is not native to the implementing team, so frontline implementers may want to consider contracting this expertise.

#### Plan to Transition Ownership to Stakeholders

One of the last steps in sustaining EBI implementation is transitioning ownership from the MIP project teams to the stakeholder(s) at the community organization where the EBI has been implemented. This includes developing transition plans with implementers at their site and identifying key personnel, including those who would review outcomes and measures, be able to realign the project if necessary, and house and disseminate training tools when workforce turnover occurs. Pulling from the work done in other stages outlined in the Implementation Roadmap, research partners, community sites, and other stakeholders can work together to ensure a viable plan for ownership transition is in place in order to support the future of these projects.

Stakeholders at MDHHS are also invested in the sustainability of these programs beyond the Medicaid matched funding mechanism. Partners at MDHHS have said that they want projects that have a sustainable funding plan; utilizing these relationships to transition financial ownership is a key step in garnering longer-term sustainability for successful projects. For example, if pursuing billing codes for specific services, teams need to link up with the correct people from the beginning to pursue this funding route. MIP is uniquely situated in that these projects can connect with key personnel in the government of the State of Michigan because of the partnership with MDHHS. This mechanism may make it easier for options like approving to fund (or pay for) certain billing codes to be pursued through the relationships this partnership affords (34). Additionally, the State of Michigan has already demonstrated their support through its decision to fund these programs and may be more able to help sustain the delivery of these programs through alternative funding mechanisms.

## Discussion

Sustainable implementation with fidelity is a difficult process (35); while knowledge of implementation science does not necessarily make implementation easier, it can help frontline implementers demystify the process by helping them prepare for potential barriers, understand strategies for addressing barriers, and structure long-term thinking (5, 6, 36, 37). As with many other academic-driven implementation efforts, MIP investigators and study teams are often leading experts in EBI development and testing, but have less expertise in transitioning their EBI to community-based practice and ownership.

The MIP Implementation Roadmap was primarily developed to enhance the practice of implementation, based in part on the extensive work by implementation researchers to develop comprehensive frameworks on the implementation process (38). The Roadmap also builds upon the emerging implementation strategy literature, which has significantly advanced much of the “how to” of implementation, specifically in developing and defining theoretically-informed frameworks for identifying determinants of implementation success as well as strategies for improving EBI uptake. Notably, the Veterans Health Administration's QUERI Implementation Roadmap was developed to demystify implementation science and encourage active partnerships between implementers, health care leaders, and practitioners (13). The MIP Roadmap intends to accomplish similar goals with a specific focus on the challenges facing non-implementation scientists, both researchers and community partners, working in community-based settings. While the MIP Implementation Roadmap is not a one-size-fits-all solution to implementation, it can help investigators navigate the steps common to implementation efforts and anticipate potential barriers, pain points, or missteps. For MIP implementation projects, which are often funded for a single year, efficient use of time is critical for meeting implementation goals. Our creation of the MIP Implementation Roadmap was motivated by a desire to assist new investigators with the design and evaluation of their community-based implementation efforts.

Although the MIP Implementation Roadmap is presented as a one-way progression, it is not necessarily meant to be followed linearly. Projects may need to address Roadmap stages at different times and certain steps or stages, such as rapid cycle testing of a tailored EBI, may need to be revisited frequently. Keeping that in mind, the MIP Implementation Roadmap may be more easily seen as a tool to guide implementers through iterative cycles of planning, experimenting, reflecting, and refining until the goals of their projects are reached.

In interviews with all MIP project teams and community partners, participants were asked to identify actions they took that were most helpful to their EBI implementation. This helped define the “best practices” they utilized to achieve successful implementation of their EBI in their community setting. Oftentimes, this conversation quickly turned to lessons learned from unexpected barriers teams encountered and the methods they deployed to overcome these challenges. Although each team identified unique problems and solutions, there were similarities to the types of challenges teams encountered. By incorporating these shared experiences into the stages outlined in the Implementation Roadmap, future MIP projects may be able to proactively avoid common barriers and be better equipped to address the challenges that are commonly faced with implementing these types of EBIs in community settings.

When interviewing both research teams and community partners, implementers did not always think of the planning and preparation they were doing as part of the implementation process. The Roadmap's elucidation of these steps as comprising the key pre-implementation phase is intended to encourage implementers to tie these planning efforts more explicitly to the goals of the implementation process. Indeed, interviews identified the largest number of barriers and best practices pertaining to the pre-implementation phase than to other phases. Although the steps taken in this phase were not necessarily considered “implementation” by teams, interviews revealed they were important to projects' successes and often required the most time and energy from project stakeholders. By specifically defining the pre-implementation phase on the Roadmap, the hope is that teams will have guidance on ways to set themselves up for success, and view these steps as integral to the implementation effort as a whole.

Although conversations around pre-implementation and implementation phases were focused on actions MIP projects had completed or were currently completing, conversations around sustainability were often hypothetical, as most MIP projects had not yet reached this stage at the time of their interviews. However, by talking with teams and investigators about what they planned to do or wish they had done sooner, we were able to understand the key challenges facing MIP project sustainability and identify steps or considerations for teams even as they begin implementation efforts. While the MIP Implementation Roadmap is currently more pertinent to the active practice of implementation, this tool can guide implementers to consider sustainability-related issues and actions earlier on in the process, ideally providing early scaffolding to support long-term implementation sustainability of EBIs.

The main threat to sustainability of most implementation projects is securing funding beyond an initial grant or funding allocation. As discussed in the sustainability phase, one of the main steps identified by MIP teams for securing a funding source beyond the year of funding guaranteed by MIP was working to develop sustainable revenue streams (e.g., “turn on” billing codes) for their particular project once positive patient outcomes had been demonstrated. Once established, this would allow practitioners at the community sites where projects are being implemented to bill insurance for the services that they are providing, thus eliminating the need for a grant funding mechanism. Although demonstrated as a viable option, many research teams noted in their interviews that they were struggling to accomplish this.

As noted in the introduction, Roadmaps in general are tools that can be used for structuring process flows. We developed this Implementation Roadmap to help guide projects through early-stage implementation efforts and beyond, regardless of a practitioner's experience in implementation. When used from a project's inception, our Implementation Roadmap can help frontline implementers systematically anticipate and address barriers to the implementation of their EBI and ensure the collection of key metrics that can aid successful implementation and sustainability; our Implementation Roadmap provides scaffolding for larger scale, sustainable implementation efforts for EBIs.

The MIP mechanism specifically affords a more direct opportunity for EBI developers and experts to implement their effective practices in communities. However, states need a guide to ensure that programs funded through the Medicaid matched mechanism are sustained at both the clinical and policy levels. This Implementation Roadmap can help in this endeavor by informing a process by which implementation occurs in community practices traditionally affiliated with large research programs. Although the MIP Implementation Roadmap was purposefully designed for intended use by future MIP programs, many of the steps and stages of implementation highlighted can be generalized to community-based implementation projects outside of the MIP partnership.

### Limitations

There were limitations faced in the creation of this Roadmap. First, this was a relatively small qualitative study involving interviews with stakeholders from six MIP community-based implementation projects for a single fiscal year. Including a larger number of MIP projects (or implementation efforts outside of MIP) may have led to identification of additional or alternative themes and/or best practices. Despite this limited population, however, the interviews covered a wide range of stakeholders representing a diverse set of six community-based implementation projects. Further, the themes and practices identified by this diverse group of stakeholders align with current knowledge in this field. Second, it is difficult to balance the specificity of individual experiences with the generalizability of recommendations designed to apply across multiple contexts. As projects focused on EBI implementation, many did not have the resources or time to develop common tools or methods such as workflow analyses, process maps, or cost estimations that could be useful in operationalizing key Roadmap components elsewhere. In the process of creating accomplishable stages to aid in the implementation process more globally, the specificity and utility of some recommendations may have been diminished or not fully captured by the Roadmap. Third, as mentioned above, conversations around sustainability were often hypothetical for projects immersed in the early implementation phase at the time of the interviews. Future work is needed to further elucidate specific strategies for sustaining EBI implementation in community-based settings. Fourth, interviews and analyses were performed by only one author. Relying on a single perspective may have introduced bias into the transcript coding and analysis process.

### Future Directions

The MIP Implementation Roadmap is designed to be a dynamic guide that may evolve based on the needs and experiences of future MIP projects and/or changes in community-based implementation environments. Future plans involve soliciting feedback from MIP investigators, sites, and MDHHS stakeholders involved with MIP, including projects that informed the initial Roadmap and new projects. The team also plans to deploy the Roadmap with future MIP investigators and evaluate the real-world use of this tool, added value to implementation efforts, ability to address implementation barriers, and utility for practitioners. Given that resources are often limited when implementing new EBIs, we also recognize that not all implementation steps included in the Roadmap may be accomplishable by all projects, and projects may prefer to know which implementation steps are more or most important for successful implementation efforts. Given our limited sample, we were unable to weight the relative importance of the implementation steps to help projects and community partners prioritize to which they would devote more or less resources in our initial work. As the team moves forward with evaluation, signals from further implementation and adaptation efforts could identify the salient core components of the Implementation Roadmap and potentially highlight a hierarchy for resonant steps within the implementation phases in future iterations of this tool. However, we believe that in its current form the Roadmap provides a tool that projects can use to begin dialogues with community partners to establish highest implementation priorities and identify steps a priori that may be challenging and/or of lower priority.

Although the MIP Implementation Roadmap was designed with MIP projects in mind, we are also actively looking for opportunities to adapt it to new clinical or organizational settings. While created for mental health projects, the steps outlined here could be applied to EBIs addressing various health outcomes in community-based contexts. While the geographical, topical, and clinical domains could be generalized to various contexts, the future expansion of the Roadmap would need to consider the unique context beyond the core elements contained in the MIP Implementation Roadmap to ensure transferability to new applications beyond MIP.

## Conclusion

The MIP Implementation Roadmap can guide MIP investigators, teams, and partners through pre-implementation, implementation, and sustainability phases of early efforts to implement EBIs, thereby improving the effectiveness of current implementation efforts and providing scaffolding for larger scale, sustainable implementation. Although specifically informed by MIP project teams and their experiences, the Roadmap provides actionable guidance beyond traditional research-to-practice frameworks relevant to the implementation of a wide variety of EBIs, including those outside of the MIP partnership or purview. By focusing on practical and user-friendly steps, the Roadmap provides a tool that helps demystify implementation for frontline clinicians, researchers, partner leadership, and community stakeholders alike, and is applicable for a range of community-based EBI implementation efforts. Using the steps outlined in the Implementation Roadmap to develop implementation plans will help establish and aid in the scale up of behavioral health programs in community settings regardless of practitioners' familiarity with implementation practice.

## Data Availability Statement

The raw data supporting the conclusions of this article will be made available by the authors, without undue reservation.

## Ethics Statement

The studies involving human participants were reviewed and approved by University of Michigan Institutional Review Board. Written informed consent for participation was not required for this study in accordance with the national legislation and the institutional requirements.

## Author Contributions

LMD, AMK, and SNS conceived and designed the MIP Implementation Roadmap project. AR collected the qualitative data, transcribed interviews, carried out the analysis, and wrote the manuscript. LMD, CML, AMK, and SNS reviewed and contributed to interpretation of findings. All authors reviewed and revised the manuscript, gave their approval of the submitted version of the manuscript, and agreed to be accountable for all aspects of the work.

## Conflict of Interest

The authors declare that the research was conducted in the absence of any commercial or financial relationships that could be construed as a potential conflict of interest.

## Abbreviations

CFIR: Consolidated Framework for Implementation Research
EBI(s): Evidence-based Intervention(s)
EPIS: Exploration, Preparation, Implementation, Sustainment
ERIC: Expert Recommendations for Implementing Change
FY: Fiscal Year
IRB: Institutional Review Board
MDHHS: Michigan Department of Health and Human Services
MIP: Michigan Mental Health Integration Partnership
PI: Principal Investigator
QUERI: Quality Enhancement Research Initiative
RE-AIM: Reach, Effectiveness, Adoption, Implementation, and Maintenance
REP: Replicating Effective Programs
UM: University of Michigan.
